# Chemical and Biological Investigations of Antiviral Agents Against Plant Viruses Conducted in China in the 21st Century

**DOI:** 10.3390/genes15121654

**Published:** 2024-12-23

**Authors:** Yuanyou Yang, Lei Hu, Tongtong Chen, Libo Zhang, Delu Wang, Zhuo Chen

**Affiliations:** 1 State Key Laboratory of Green Pesticides, Key Laboratory of Green Pesticide and Agricultural Bioengineering, Ministry of Education, Guizhou University, Guiyang 550025, China; gs.yangyy16@gzu.edu.cn (Y.Y.); 18285877610@163.com (L.H.); lbzhang@gzu.edu.cn (L.Z.); 2 College of Agriculture, Guizhou University, Guiyang 550025, China; 15186785529@163.com; 3 College of Forestry, Guizhou University, Guiyang 550025, China; dlwang@gzu.edu.cn

**Keywords:** plant virus disease, antiviral agent, chemical biology, mode of action, drug molecules, bio-targets

## Abstract

Research into the biology of plant viruses, their mechanisms of pathogenicity, and the induction of host resistance has laid a solid foundation for the discovery of antiviral agents and their targets and the development of effective control technologies. Additionally, recent advancements in fields such as chemical biology, cheminformatics, bioinformatics, and synthetic biology have provided valuable methods and tools for the design of antiviral drugs, the synthesis of drug molecules, assessment of their activity, and investigation of their modes of action. Compared with drug development for human viral diseases, the control of plant viral diseases presents greater challenges, including the cost-benefit of agents, simplification of control technologies, and the effectiveness of treatments. Therefore, in the current context of complex outbreaks and severe damage caused by plant viral diseases, it is crucial to delve deeper into the research and development of antiviral agents. This review provides a detailed overview of the biological characteristics of current targets for antiviral agents, the mode of interaction between plant virus targets and antivirals, and insights for future drug development. We believe this review will not only facilitate the in-depth analysis of the development of antivirals for crops but also offer valuable perspectives for the development of antiviral agents for use in human and veterinary medicine.

## 1. Introduction

Anti-plant virus agents encounter difficulties in exerting their effects due to the obligately parasitic nature of plant viruses and the specific tissue and cellular structures of plants, such as the waxy epidermal layer on leaf surfaces. To date, in contrast to fungicides, insecticides, and herbicides, the variety of antiviral agents against plant viruses developed both domestically and internationally remains relatively limited, with a narrow range of types and a limited number of viral targets and target viruses [1]. In recent years, changes in farming patterns have led to large-scale monoculture of crops, as well as the emergence of viruses that spread through seed transmission, mechanical transmission, and other means. Additionally, global warming and the long-term irrational use of pesticides—resulting in increased pest resistance and difficulties in pest (and virus) control—have contributed to a year-on-year escalation in the incidence of crop viral diseases, causing substantial economic losses to agricultural production [1,2]. Therefore, the development of effective antiviral agents is urgently required to address the challenges of viral disease control in agriculture [1]. There are numerous types of plant virus diseases, diverse transmission pathways, and complex patterns of prevalence [2]. The majority of viral diseases are latent and have no obvious symptoms in the early stages, but they cause significant harm in the later stages [2,3]. Due to the challenges in preventing and controlling viral diseases, plant viral disease is often referred to as “plant cancer” in agriculture. Farmers mainly employ strategies such as selecting disease-resistant varieties, improving cultivation methods, utilizing insecticides to control vector insects, and other approaches [2,3]. Anti-plant virus agents can be categorized into three types based on their mode of action: viral inactivation agents (which destroy the viral particle), virus curative agents (which inhibit the virus), and plant activators against the virus (which enhance host resistance) [1,4,5]. The methods for applying these three types of antiviral agents differ in agricultural practice. In the early stages of viral disease or during the seedling phase of crops, the primary objectives are to enhance host resistance, alleviate plant symptoms and damage, and reduce the viral load within the host using plant activators. During the infection period, viral inactivation agents are primarily employed to destroy the virus and mitigate the risk of further infection. When significant viral proliferation occurs, the focus shifts to using viral curative agents to inhibit the replication and spread of the virus [1,2,3].

In recent years, China has gradually emerged as the world’s largest producer of crop protection chemicals. In response to the challenging issue of controlling plant virus diseases, the country has initiated research and development efforts focused on antiviral agents for plants. Since the beginning of this century, significant progress has been made in the study of anti-plant virus agents. Notable advancements include research into the targets of plant viruses and the mechanisms for inducing disease resistance in hosts [6], the design of molecules based on these viral targets [7], and the discovery of new natural products with antivirus activity derived from plants [8,9,10], animals [6], microbes [11], and marine organisms [12].

However, the creation of antiviral agents for use on plants is quite challenging due to several factors, including the cost of the agent, their environmental risk, field application technology, control effectiveness, crop type, and field application contexts [1,3]. The development of antiviral agents for use on plants differs fundamentally from that of medicinal and veterinary drugs; thus, it cannot simply rely on the research and development approaches used in those fields. It is essential to summarize the innovative concepts surrounding the development of antiviral agents for plants in China over the past 20 years. This includes the discovery and rational evaluation of targets, design principles for antiviral agents for plants, and lead molecules. Systematic thinking is crucial in integrating these elements to advance research and development in this area. This paper focuses on several key aspects: the druggability of plant virus targets, the interaction modes between targets and active substances, the effects of anti-plant virus substances, the research models for anti-plant virus targets, and the screening methods for anti-plant virus agents. Although plant activators play a significant role in combating plant viruses, this paper will not review work in this field due to space limitations. For more information, please refer to our review on plant activators [5].

## 2. Antiviral Active Substances Targeting Plant Virus Components and Their Modes of Action

### 2.1. Viruses and Potential Targets of Plant Antiviral Agents

#### 2.1.1. Tobacco Mosaic Virus (TMV)

**Figure 1:**
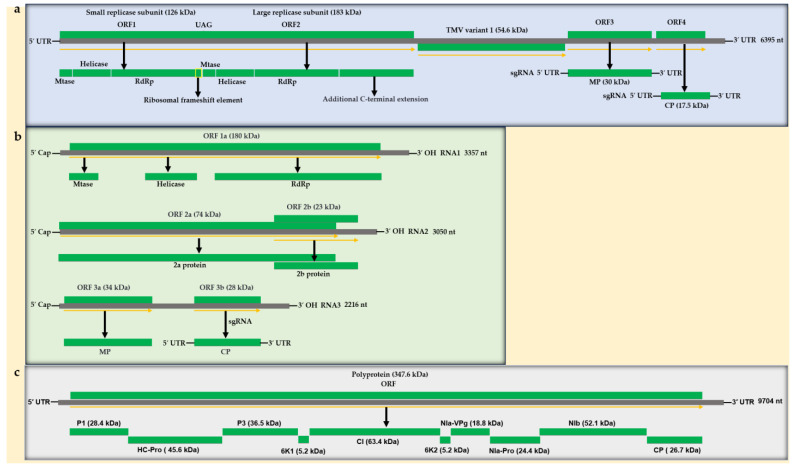
Genome organization and encoded proteins of tobacco mosaic virus (TMV) (**a**), cucumber mosaic virus (CMV) (**b**), and potato virus Y (PVY) (**c**). kDa, kilodalton; ORF, open reading frame; Mtase, methyltransferase; RdRp, RNA-dependent RNA polymerase; MP, movement protein; CP, coat protein; sgRNA, subgenomic RNA; UTR, untranslated region; 5′cap, the cap structure at the 5′ end of RNA; 3′OH, the hydroxyl (OH) group at the 3′ end; P1, protein 1; HC-Pro, helper-component proteinase; P3, protein 3; 6K1, 6K1 protein (6 kDa); 6K2, 6K2 protein (6 kDa); CI: cytoplasmic inclusion; NIa-VPg, NIa protein-viral protein genome-linked; NIa-Pro, NIa proteinase; Nib, NIb protein. The complete genome of TMV and PVY are available from the NCBI Reference Sequence (TMV, NCBI Reference Sequence: NC_001367.1, BioProject: PRJNA485481; PVY, NCBI Reference Sequence: NC_001616.1, BioProject: PRJNA485481). The complete sequences of RNA 1, RNA 2, and RNA 3 of CMV are provided under NCBI (RNA 1, NCBI Reference Sequence: NC_002034.1, BioProject: PRJNA485481; RNA 2, NCBI Reference Sequence: NC_002035.1, BioProject: PRJNA485481, and RNA 3, NCBI Reference Sequence: NC_001440.1, BioProject: PRJNA485481).

TMV is a positive-sense single-stranded RNA virus that can infect a wide range of plants, including solanaceous plants. The wild-type TMV has a hollow tubular structure measuring 300 nm in length, with outer and inner diameters of 18 nm and 4 nm, respectively. It consists mainly of a protein shell, which makes up 95.0% of its total mass, while the remaining 5.0% is made up of genomic RNA [13,14,15]. The total length of the genomic RNA of TMV is 6395 nucleotides and consists of various regions, such as a 5′ untranslated region, a *replicase* gene, a *movement protein* (*MP*) gene, a *coat protein* (*CP*) gene, and a 3′ untranslated region. The CP is particularly important to plant virologists due to its role in virus assembly. It can trigger virus-specific host resistance in transgenic virus-resistant plants. The protein shell is made up of 2132 identical CP subunits arranged in a right-handed helical spiral that encloses the single-stranded RNA. Each protein subunit has an affinity for three nucleotides, and there is an average of 16.3 subunits per revolution, resulting in a total of 131 helices. The TMV CP monomer contains 158 amino acid residues with a molecular weight of 17.5 kDa. Notably, both the N-terminus and C-terminus of the CP are located on the outer surface of the nanotube structure (Figure 1) [16,17].

At present, researchers in China are focused on developing antiviral agents that target the CP. The 126 kDa protein encoded by the gene includes sequence motifs common to methyltransferases and helicases, whereas the 183 kDa protein features motifs typical of RNA-dependent RNA polymerases (RdRp). Both proteins are essential for the effective replication of TMV RNA [18]. The replication mechanism of plant viruses is also a potential target for anti-plant virus agents, and in recent years, research has been conducted in this area. TMV has played a pivotal role in establishing foundational theories in viral biology and antiviral agents, providing essential insights into the structural properties, composition, and replication mechanisms of viruses, as well as the typical symptoms of plant viral diseases [19]. When TMV particles enter a host cell, the virions quickly disassemble in a process involving destabilization, likely caused by the repulsive forces between carboxyl-carboxylate groups on amino acids at positions 50 (glutamic acid; Glu, E) and 77 (aspartic acid; Asp, D). These amino acids are situated at the interface of adjacent CP subunits. In the extracellular environment, the negative charges on these carboxylate groups are stabilized by cations like Ca^2+^ or protons [20]. Understanding the components of TMV and the corresponding processes in plants would aid in the establishment of models for antiviral agents and subsequent research. To date, TMV RNA, subgenomic RNA (sgRNA), CP, helicase, and helper-component proteinase (HC-Pro) have been utilized as targets for antiviral agents, and their modes of action with these agents have been studied (Figure 1a).

#### 2.1.2. Cucumber Mosaic Virus (CMV)

As a member of the *Cucumovirus* genus in the *Bromoviridae* family, Cucumber mosaic virus (CMV) can infect a broad spectrum of plant species. The viral genome consists of three RNA segments, which together encode five distinct proteins. RNA 1 is the sole monocistronic RNA and encodes the 1a protein, which plays a crucial role in viral replication. This protein contains functional motifs for methyltransferase and helicase activities [21,22]. RNA 2 is responsible for encoding the 2a protein, an essential component in viral polymerase function [23,24]. RNA 4A, a low-abundance subgenomic RNA (sgRNA), encodes the 2b protein, which functions as a viral suppressor of RNA silencing [25]. The 2b protein, among the earliest known suppressors of RNA silencing, can function at various stages of the silencing process. It interacts with Argonaute 1 (AGO1) and AGO4 to inhibit their cleavage activities [26,27]. The 5′ open reading frame (ORF) from RNA 3 encodes an MP, while the sgRNA 4 encodes the CP [28]. The latter two proteins are essential for viral movement (Figure 1b) [28]. To date, CMV CP and 2b proteins have been used as targets for antiviral agents, and their modes of action with these agents have been studied.

#### 2.1.3. Potato Virus Y (PVY)

PVY, a member of the genus *Potyvirus* within the family *Potyviridae*, has a positive-sense, single-stranded, linear RNA genome [29]. The genome includes a single ORF that encodes a polyprotein consisting of 3061 amino acids [30]. The viral polyprotein is converted into nine mature peptides through the action of the virus-encoded proteinase [31]. The reading frame of the *P3* gene undergoes a +2 frameshift to produce an additional viral protein [32]. PVY can cause infections in a wide range of solanaceous crops, such as potato, pepper, tomato, and tobacco, resulting in substantial decreases in crop production [33,34,35]. The CP plays several essential roles in the PVY virus lifecycle, maintaining the stability and conformation of viral particles, assisting in their assembly, and facilitating the formation of virus-like particles (VLPs), which depend on the N-terminus. Additionally, the CP supports viral RNA replication, stability, intercellular movement, and long-distance transport of the virus. Research indicates that changes or deletions in the N- and C-termini can significantly affect the virus’s systemic infection capability. These functions highlight the CP of PVY as a key target for understanding viral pathogenesis and developing antiviral agents [36]. The HC-Pro protein of PVY is involved in the processing of the polyprotein, the movement and transmission of the virus within the host, and the suppression of host antiviral RNA silencing [37,38,39,40]. The viral genome-linked protein (VPg) exhibits RNA silencing inhibitory activity and is crucial for the virus’s cell-to-cell movement and long-distance transport (Figure 1c) [41,42]. In recent years, the CP, VPg, and HC-Pro of PVY have increasingly been selected as targets for antiviral agents to study their modes of action and aid in the development of novel antiviral agents.

#### 2.1.4. Telosma Mosaic Virus (TeMV)

TeMV, a member of the genus *Potyvirus*, has a near 9.7–15.8 kb single-stranded RNA genome, with its 5′ end covalently linked to the VPg protein, and is present at a high incidence in the main passion-fruit growing regions of China [43,44,45]. At present, the complete genome sequences and functions of TeMV are not clearly defined. P1, the first protein translated from most potyvirus genomes, includes an N1 domain that is vital for viral infection due to its role in regulating the abundance of its cognate HC-Pro [46]. Research indicates that the VPg protein of TeMV, which is covalently linked to the viral genome, plays a crucial role in viral replication by interacting with the cap-binding translation initiation factor eIF4E [47]. In recent years, the VPg of TeMV has been targeted for the design of antiviral agents to study their mode of action. We will also review it separately in this article.

#### 2.1.5. Tomato Spotted Wilt Virus (TSWV)

TSWV, a representative member of the order *Bunyavirales*, the family *Tospoviridae*, and the genus *Orthotospovirus*, can cause significant economic losses in numerous crops worldwide [48,49]. The virions of TSWV are membrane-bound particles with diameters ranging from 80 to 120 nm. The virus contains a single-stranded RNA genome divided into three segments: large (L), medium (M), and small (S), based on their size. The L segment encodes the RNA-dependent RNA polymerase (RdRp) (∼330 kDa) on the viral complementary (vc) RNA. The M segment, which has an ambisense polarity, encodes a non-structural movement (NSm) protein on the viral (vRNA) strand and a precursor protein for the glycoproteins (Gn and Gc, with n and c, referring to the amino- and carboxy-terminal positions within the precursor) on the vcRNA strand. The S segment, also with ambisense polarity, encodes the non-structural protein (NSs) on the vRNA strand, which serves as a suppressor of RNA silencing, and the nucleocapsid (N) protein on the vcRNA strand [50,51]. The NSs play a crucial role in the infection, replication, and transmission of TSWV [52,53,54]. 

For instance, the NS protein suppresses post-transcriptional gene silencing in plants and disrupts RNA silencing in arthropod cell lines [53,54]. The N protein is the main structural component of the virus, forming a protective shell around the viral genome and creating ribonucleoprotein complexes (RNPs) with RdRP and RNA [55,56,57]. It also facilitates the virus’s movement within and between cells by interacting with the glycoproteins Gn and Gc [58,59]. Therefore, the NSs and N proteins have been selected as targets for antiviral agents to study their modes of action and to aid in the development of these agents.

#### 2.1.6. Southern Rice Black-Streaked Dwarf Virus (SRBSDV)

Rice is a major food crop, and viral diseases can significantly reduce yields, posing major threats to food security. SRBSDV is a non-enveloped icosahedral virus with a genome of ten double-stranded RNA segments. Classified as a new species in the *Fijivirus* genus (*Reoviridae* family), it is transmitted by the white-backed planthopper (WBPH, *Sogatella furcifera*) in a persistent, circulative, and propagative manner [60,61]. SRBSDV can also infect maize, Job’s tears (*Coix lacryma-jobi*), and various grass weeds through WBPH. Infected rice plants show symptoms similar to those caused by the Rice black-streaked dwarf virus (RBSDV). Early symptoms in rice may be subtle, but later-stage symptoms can severely affect yield, with stunting and plant death following seedling infection [62]. Control measures primarily involve early monitoring, the development of disease-resistant varieties, and the integrated use of pesticides, along with prevention and treatment strategies for viral diseases [3,63,64]. P9-1, which represents a similarity with the corresponding proteins in RBSDV, serves as a viroplasm protein and plays a role in virus replication and assembly [65]. An assay based on size-exclusion chromatography revealed that P9-1 can self-assemble into octamers in vitro. Mutation analysis of the terminal amino acids suggests that the C-terminal region (amino acids 324–347) is crucial for octamer formation. Structural predictions and yeast two-hybrid experiments further support the critical role of the C-terminal arm of P9-1 in facilitating octamer assembly and maintaining the viroplasm structure through self-interaction [66]. This insight is crucial for the advancement in antiviral strategies. Given the amino acid homology between SRBSDV P10 and RBSDV P10, it is inferred that SRBSDV P10 serves as the CP of the virus and is vital for the virus’s ability to spread and infect host plants [67,68]. SRBSDV P10 is considered a promising target for managing SRBSDV. Protein P10 has been successfully cloned and expressed, and the protein was isolated from *Escherichia coli*. The binding of ferulic acid derivatives to P10 has been confirmed using techniques including size-exclusion chromatography, fluorescence titration (FT), and microscale thermophoresis (MST), revealing a high-affinity interaction. 3D-quantitative structure–activity relationship (QSAR) studies, both ligand- and receptor-based, suggested that carboxyl groups interact with P10 [69]. The expression and purification systems developed, along with the methods for probing drug–P10 interactions, serve as valuable tools for evaluating the efficacy of antiviral compounds against SRBSDV. Consequently, P9-1 and P10 are key targets in the study of the modes of action of antiviral agents.

### 2.2. The Mode of Action of Viral Components and Plant Antiviral Agents

**Figure 2:**
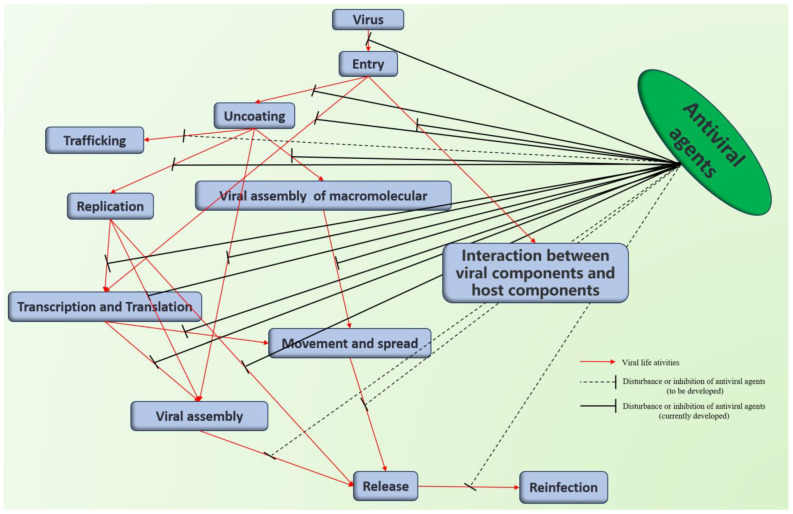
The mode of action of plant antiviral agents against viral components.

Currently, the development of antiviral agents for use on plants is aimed primarily at TMV and CMV. In recent years, efforts have also been made to expand this focus to include PVY, SRBSDV, Pepper mottle virus (PepMoV), TeMV, TSWV, and other viruses. The main viral targets are mainly the CP, as well as the helicase and HC-Pro proteins. However, there has been little development of antiviral agents targeting nucleic acids (Figure 2). The research teams from Nankai University, China, have conducted some novel work on the development of antiviral agents. Regarding the target of TMV CP, current studies suggest that the agents primarily operate by interfering with virus assembly. We have categorized the sources of leading structures for antiviral agents into chemical synthesis, botanical sources, microbial sources, animal sources, and marine biological sources. Chemical synthesis is the primary method for creating antiviral agents, and there are currently two strategies: first, rational design based on the target, although relatively little work has been conducted on the design and synthesis of active molecules for antiviral agents targeting plant viruses; and second, identification of lead compounds from natural products. Guizhou University, Nankai University, and other institutes in China have conducted extensive research into antiviral agents for plants. In the following subsections, we will describe the recent research in this field on specific components, particularly nucleic acids, of major plant viruses.

#### 2.2.1. TMV RNA

**Figure 3:**
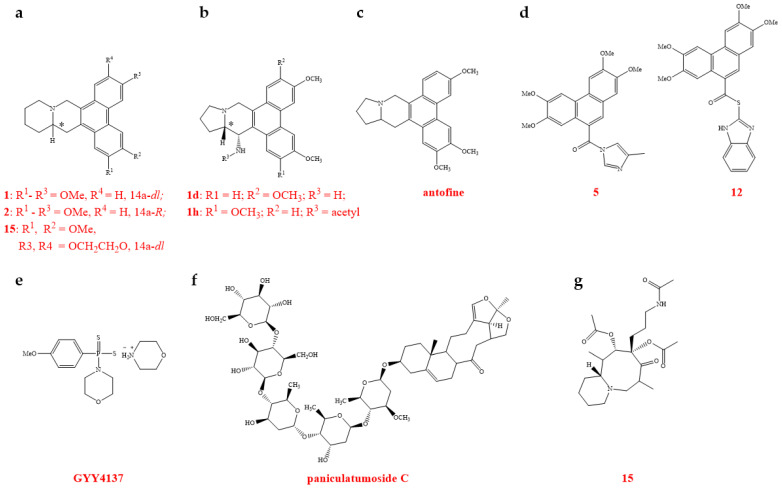
Structures of some compounds with anti-TMV activity that act on the RNA of TMV. (**a**) Phenanthroquinolizidine alkaloid derivatives containing a phenanthrene moiety (**1**, **2**, **15**, and **16**), (**b**) 14-aminophenanthroindolizidine derivatives (**1d** and **1h**), (**c**) antofine, (**d**) phenanthrene-containing *N*-heterocyclic compounds containing the parent structure of antofine (**5** and **12**), (**e**) H_2_S donor **GYY4137**, (**f**) paniculatumoside C, and (**g**) an aloperine-type alkaloid (**15**).

Phenanthroquinolizidine alkaloids were synthesized and evaluated for their antiviral efficacy against TMV using a half-leaf bioassay technique. Alkaloids with substituents on the phenanthrene ring (compounds **1**, **2**, **15**, and **16**) exhibited strong antiviral effects in vivo at a concentration of 500 µg/mL, with mean ± standard deviation (SD) efficacy rates of 71.5% ± 2.0, 76.2% ± 2.0, 66.6% ± 1.0, and 77.3% ± 3.0, respectively. These rates surpassed that of ningnanmycin (NNM), which had an antiviral efficacy of 56.0 ± 2%. Moreover, the addition of a 6-hydroxyl group to the molecule was found to enhance its interaction with TMV RNA, thereby improving its antiviral activity against TMV (Figure 3a, Table 1) [70]. Additionally, a series of 14-aminophenanthroindolizidine was synthesized, and their anti-TMV activity was determined using a bioassay. The candidate compounds **1d** and **1h** exhibited excellent anti-TMV activity in vivo at a concentration of 500 µg/mL, with antiviral activities of 68.0% and 69.6%, respectively. These activities were also superior to that of NNM, which showed an antiviral activity of 56.0%. The incorporation of amino groups at position C-14 of phenanthroindolizidines enabled interaction with the TMV RNA, thereby boosting anti-TMV efficacy (Figure 3b, Table 1) [71].

**Figure 4:**
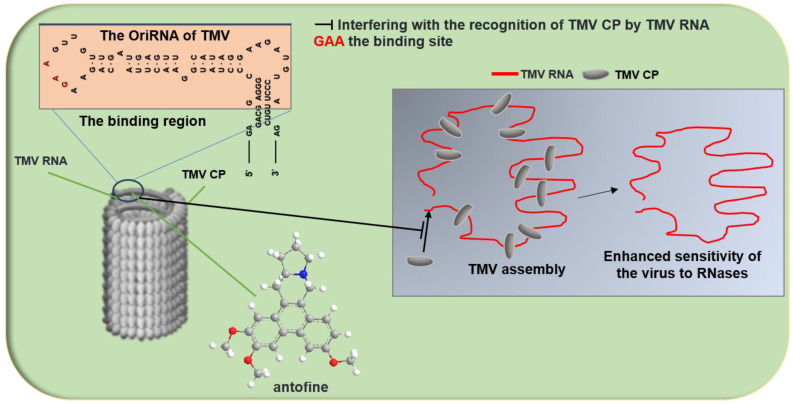
The mode of action between antofine and the RNA of TMV. CP, coat protein; TMV, *Tobacco mosaic virus*; OriRNA, assembly origin of TMV RNA.

Antofine or 7-demethoxylophorine is a component isolated from the Chinese medicinal plant *Cynanchum komarovii* from the family Asclepiadaceae. It has been shown that antofine exhibits inhibitory activities against both TMV and PVY. The site where antofine acts on TMV RNA, specifically on bulged structures, may be located at the hairpin structure at the 5′ end of the RNA (Figure 3c and Figure 4). This interaction interferes with the recognition of CP by TMV RNA, thereby inhibiting the assembly of the virus. Consequently, TMV RNA is degraded and destroyed by the nuclease present in the plant host, leading to the loss of the virus’s infectivity (Table 1) [72,73]. A random library of TMV RNA assembly start sites was constructed, and a random screening method based on the TMV RNA assembly sequence was developed to evaluate the effects of antiviral agents on TMV assembly [73]. A range of antofine derivatives with specific binding affinities to TMV RNA was synthesized to assess the impact of antofine on TMV RNA. The findings showed that antofine and its derivatives can attach to TMV RNA and interfere with viral assembly in vitro. Additionally, the studies with antofine analogues confirmed that antofine binds preferentially to the bulged regions of TMV RNA (Table 1) [74]. Given antofine’s robust affinity for TMV RNA, it is imperative to refine the structure of the active compound to generate more effective anti-TMV agents and to lower the synthesis costs. A set of phenanthrene-based *N*-heterocyclic compounds was created. Compounds **5**, **12**, and **21** showed anti-TMV efficacy comparable to or exceeding that of antofine. Fluorescence spectroscopy analysis of these compounds with TMV RNA revealed that compounds **5** and **12** interacted with TMV RNA differently compared to antofine (Figure 3d). This discovery expands the range of potential plant antivirus targets related to structural analogues of antofine (Figure 4) (Table 1) [75].

RNA viruses that infect plants can induce the production of H_2_S, which is considered a defensive substance against viral invasion. Therefore, the development of molecules containing H_2_S with similar structures holds promise for creating novel antiviral agents. Studies have shown that the H_2_S donor **GYY4137** can significantly inhibit the replication of viruses such as TMV (Figure 3e). Additionally, it reduces the gene expression of the genes encoding RdRp and CP [76].

**Table 1:** The summarizing of antiviral agents with anti-TMV activity that act on the TMV-RNA.

Antiviral Agents	Sources	Mechanisms	Efficacy ^a, b^	References
Phenanthroquinolizidine alkaloids **16**	Synthesis	Binds RNA	77.3%	[70]
14-aminophenanthroindolizidines **1h**	Synthesis	Binds RNA	69.6%	[71]
Antofine	Extraction	Binds oriRNA of TMV	60.0% at 1.0 µg/mL	[73]
Antofine analogues **2**	Synthesis	Binds RNA, therefore disrupting virus assembly	Assembly inhibition efficiencies: 90.7%	[74]
Phenanthrene containing *N*-heterocyclic compounds **12**	Synthesis	Binds RNA	56.0%	[75]
Paniculatumoside C	Extraction	Inhibits CP *sgRNA* expression, therefore reducing viral replication	93.0% at 40 nM	[77]
Aloperine-type alkaloids **4**	Synthesis	Inhibits transcription of *CP* gene	67.7% at 100 μg/mL	[78]

^a^ If the specific concentration is not listed in the ‘Efficacy’ column, the concentration is assumed to be 500 μg/mL. ^b^ If the specific mode of action is not listed in the ‘Efficacy’ column, the mode of action is assumed to be curative activity.

#### 2.2.2. TMV sgRNA

The components from the plant *Strobilanthes cusia* (Assam indigo) were separated and identified. The *seco*-pregnane steroid glaucogenin C and its glycosides exhibit potent antiviral effects against TMV, *Sindbis virus* (SINV), *Getah virus* (GETV), and *Eastern equine encephalomyelitis virus* (EEEV). Paniculatumoside C, in particular, can suppress the expression of the *CP* gene, thereby hindering the virus’ ability to spread systemically, decreasing TMV replication, and minimizing the overall viral infection. Additionally, it was found that paniculatumoside C had no effect on the genomic RNA of TMV, further confirming that its mechanism of action involves the inhibition of *CP* sgRNA expression (Figure 3f, Table 1) [77]. The components of *Sophora alopecuroides* seeds were isolated and identified, and aloperine-type alkaloids were evaluated for their antiviral activity against TMV. Compounds **4**, **15**, **20**, and **21** showed strong protective and curative activities in vivo at 100 µg/mL, with antiviral activities of 67.7% ± 2.2, 64.6% ± 2.7, 65.3% ± 2.1, and 67.3% ± 1.2, respectively, outperforming NNM at 64.3% ± 1.2. Compound **15**, in particular, significantly inhibited *CP* gene transcription (Figure 3g, Table 1) [78].

#### 2.2.3. TMV CP

##### Disrupting the Assembly of the TMV CP

(a) Botanical sources

**Figure 5:**
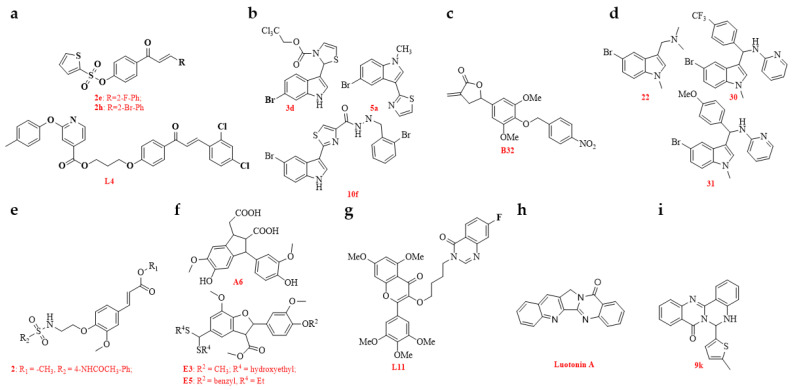
Structures of some lead compound derivatives from plants with anti-TMV activity that disrupt the assembly of TMV CP. (**a**) Chalcone derivatives containing a thiophene sulfonate group or phenoxypyridine moiety (**2e**, **2h**, and **L4**), (**b**) camalexin derivatives (**3d**, **5a**, and **10f**), (**c**) an α-methylene-γ-butyrolactone derivative **B32**, (**d**) gramine structural analogues (**22**, **30**, and **31**), (**e**) ferulic acid ester containing sulfonamide moieties (**2**), (**f**) ferulic acid derivatives containing oxime ether or dithioacetal groups (**A6**, **E3**, and **E5**), (**g**) a myricetin derivative containing a quinazolinone moiety (**L11**), (**h**) luotonin A, and (**i**) a luotonin A derivative (**9k**).

Currently, most antiviral lead compounds are derived from plants. Chalcone is recognized for its potent antimicrobial properties, and novel chalcone derivatives, featuring either a thiophene sulfonate group or a phenoxypyridine moiety, were synthesized and assessed for anti-TMV activity. Among them, compounds **2e**, **2h**, and **L14** demonstrated significant anti-TMV effects (Figure 5a). Additionally, compound **2e** exhibited remarkable inactivation activity against TMV, with a half-maximal effective concentration (EC_50_) value of 44.3 µg/mL, surpassing that of NNM (120.6 µg/mL). MST analysis showed that the binding of compounds **2e** and **2h** to TMV CP yielded equilibrium dissociation constant (K_d_) values of 0.270 and 0.301 µmol/L, respectively, which are superior to that of NNM (0.596 µmol/L). Molecular docking (MD) studies of **2e** and **2h** with TMV CP revealed that these compounds were well-embedded in the pocket between the two subunits of TMV CP (Table 2) [79,80].

The development of effective antivirals from plant allelochemicals is an important approach. Analogues based on the structure of the phytoalexin camalexin were synthesized and evaluated for antiviral activity. Of these, camalexin derivatives **3d**, **5a**, and **10f** exhibited high anti-TMV effects in vivo, surpassing both ribavirin and NNM (Figure 5b). Compound **5a** was shown to promote the fusion of 20S CP disks, interfering with virus assembly. MD simulations revealed multiple hydrogen bonds and strong binding interactions between TMV CP and several candidate camalexin-derived compounds. The binding affinities of compounds **5a** and **10f** for TMV CP were further confirmed by FT analysis (Table 2) [81]. The authors believe this work is significant in the search for novel lead compounds for antiviral agents.

Based on the structure of nicotlactone A, a series of novel α-methylene-γ-butyrolactone derivatives was designed and synthesized, and their antiviral activities were evaluated. Compound **B32** exhibited high anti-TMV activity in vivo at a concentration of 500 µg/mL, with an inactivation rate of 88.9%, a protective rate of 65.8%, and a curative rate of 52.8%. These results significantly surpassed those of the commercial virucides ribavirin, which exhibited rates of 54.5% ± 2.2, 45.8% ± 2.1, and 42.6% ± 2.5, respectively, and NNM, with rates of 78.8% ± 2.5, 62.6% ± 1.6, and 53.8% ± 3.3, respectively (Figure 5c). Compound **B32** had the capability to compromise the structural integrity of viral particles and exhibited a robust binding interaction with the TMV CP, as indicated by MD simulations and isothermal titration calorimetry (ITC) experiments. The researchers proposed that compound **B32** might disrupt the self-assembly process of TMV through its interaction with the TMV CP (Table 2) [82]. Gramine, an indole alkaloid prevalent in barley or other plant species [83], and its structural derivatives were synthesized, and their anti-TMV potential was assessed. Compounds **22**, **30**, and **31** exhibited superior in vivo antiviral activity compared with ribavirin and NNM (Figure 5d). The authors proposed that these compounds may serve as novel leads in the development of anti-TMV agents. Additionally, these compounds represent a new antiviral mechanism, likely inhibiting the assembly of TMV by cross-linking TMV CP, thereby affecting the virus’s activity (Table 2) [84].

Ferulic acid esters containing sulfonamide groups were synthesized and evaluated for antiviral activity. Compounds **2**, **6**, **7**, **12**, and **14** demonstrated strong anti-TMV effects in vivo at a concentration of 500 µg/mL, with efficacy ranging from 39.8% to 57.9%, surpassing the control antiviral drug ribavirin, which exhibited 49.3% efficacy. In particular, compound **2** formed stable hydrogen bonds with TMV CP amino acid residues Asp219, Gln257, Ser255, Asn73, Tyr139, and Ser138 (Figure 5e). These discoveries offer essential insights into the development of antiviral compounds, presenting novel molecular designs and interaction pathways with which to fight TMV (Table 2) [85]. Biopolymers derived from ferulic acid, incorporating antiviral substructures such as oxime ether and dithioacetal, were designed, synthesized, and evaluated for their ability to inhibit TMV. Several compounds, including **A6**, **E3**, and **E5**, demonstrated strong in vivo TMV inhibition at a concentration of 500 µg/mL, with efficacy rates of 52.8% ± 1.9, 45.1% ± 1.6, and 52.6% ± 2.3, respectively. These rates outperformed those of ferulic acid (36.6% ± 3.1), ribavirin (33.5% ± 3.1), and NNM (51.5% ± 2.3) (Figure 5f). Transmission electron microscopy (TEM) and self-assembly studies revealed that compound A6 compromised the structural integrity of TMV particles. The MST assay demonstrated that compounds **A6**, **E3**, and **E5** exhibited strong binding affinity to the TMV CP. MD simulations showed that compound A6 interacted with TMV CP through multiple hydrogen and hydrophobic interactions, resulting in a change in the TMV CP configuration (Table 2) [86].

Myricetin derivatives exhibit broad bactericidal activities, and their potential for anti-plant virus activity has also been explored. Myricetin derivatives incorporating a quinazolinone moiety were designed, synthesized, and evaluated for antiviral activity. Two candidate compounds, **L11** and **L13**, showed enhanced antiviral effects in vivo at 500 µg/mL, with inhibition rates of 63.1% ± 1.0 and 57.9% ± 3.6, respectively, surpassing the efficacy of NNM (54.1% ± 2.1) (Figure 5g). The MST assay revealed a high binding affinity of compound **L11** for TMV CP, with a K_d_ of 0.012 µM (Table 2) [87].

Luotonin A was isolated from the Chinese herbal medicinal plant *Peganum nigellastrumBunge* [88]. The luotonin A alkaloid possesses plant antivirus properties and is considered a promising candidate for the development of antiviral agents. Luotonin A analogues were designed, synthesized, and tested for efficacy against TMV. The study revealed that several of these derivatives, including compounds **9k**, **12b**, and **12d**, exhibited enhanced antiviral effects against TMV in vivo compared with ribavirin (Figure 5i). TEM and MD simulations demonstrated that compound **9k** could form hydrogen bonds with TMV CP, leading to the polymerization of CP and thus obstructing viral assembly and infection. This and other findings suggest that Chinese medicinal plants may serve as a source of active compounds with plant antivirus activity (Table 2) [89].

**Table 2:** The summarizing of chemical synthetic antiviral agents from plants with anti-TMV activity that disrupt the assembly of TMV CP.

Antiviral Agents	Mechanisms	Efficacy (EC_50_ or Inhibition Rate) ^a, b^	References
Novel chalcone derivative **2e**	Binds CP, therefore affecting viral assembly	EC_50_: 44.3 μg/mL	[79,80]
Camalexin derivative **3d**	Binds CP, therefore disrupting the viral assembly	60.0%	[81]
α-methylene-γ-butyrolac-355 tone derivative **B32**	Binds CP, therefore disrupting the structure of viral particles	88.9% (Inactivation activity); 52.8% (Curative activity)	[82]
Indole alkaloid gramine analogue **31**	Cross-links CP, therefore inhibiting viral assembly	65.0%	[84]
Ferulic acid ester containing sulfonamide moiety **7**	Bonds CP through hydrogen bond	57.9% (Curative activity)	[85]
Ferulic acid derivative with oxime ether and dithioacetal **A6**	Bonds CP, therefore affecting the structure of viral particles	52.8% (Inactivating activity)	[86]
Myricetin derivatives with a quinazolinone moiety **L11**	Binds CP	63.1%	[87]
Luotonin A derivative **12b**	Binds CP through hydrogen bond, therefore inhibiting viral assembly	54.0%	[89]

^a^ If the specific concentration is not listed in the ‘Efficacy’ column, the concentration is assumed to be 500 μg/mL. ^b^ If the specific mode of action is not listed in the ‘Efficacy’ column, the mode of action is assumed to be curative activity.

(b) Animal sources

**Figure 6:**
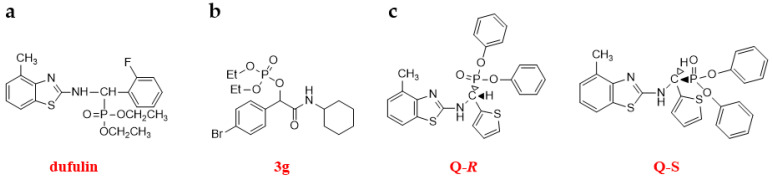
Structures of some lead compound derivatives from animal sources with anti-TMV activity that disrupt the assembly of the TMV CP. (**a**) Dufulin, (**b**) an α-amide phosphate derivative candidate compound (**3g**), and (**c**) enantiomeric α-amino phosphonate derivatives (**Q-*R*** and **Q-*S***).

Dufulin is a new antiviral agent developed by researchers at Guizhou University, China (Figure 6a). Its active component is derived from the amino phosphonate of sheep, produced by bionic synthesis, and exhibits strong control activity against TMV, SRBSDV, and other viruses. It also has activation functions and can induce the salicylic acid-signaling pathway in plant hosts, thereby enhancing their disease resistance [6]. Although less work has been conducted to develop plant antiviral agents based on target design, Guizhou University has begun this endeavor in recent years. As an example, from a library of 43,417 compounds, **SH-05** was selected as the lead compound. α-Amide phosphate analogues based on **SH-05** were developed, synthesized, and assessed for their effectiveness against TMV. Among the candidates, compounds **3g**, **3h**, and **3n** exhibited notable anti-TMV properties, both in curative and inactivation capacities in vivo at a dose of 500 µg/mL. Compound **3g** showed curative and inactivation activities of 62.9% ± 1.6 and 92.8% ± 2.7, compound **3h** showed 58.7% ± 3.1 and 89.4% ± 2.1, and compound **3n** showed 53.5% ± 3.2 and 90.0% ± 1.0, respectively; these outcomes outperformed those of NNM, which exhibited 55.4% ± 3.6 (curative activity) and 90.5% ± 1.0 (inactivation activity). MST assays indicated a strong binding interaction between compound **3g** and TMV CP (Figure 6b). MD analysis further verified that candidate compound **3g** could bind to TMV CP through hydrogen bonding, attractive charge interactions, and π-cation interactions. The TEM studies indicated that the candidate compound **3g** could disrupt the structure of TMV particles, leading to a loss of infectivity [90].

α-Amino phosphonates are recognized for their potent antiviral activity, though the mechanisms underlying this effect require further investigation. Enantiomeric α-amino phosphonate derivatives with strong anti-TMV properties were synthesized. Antiviral testing revealed that the (*R*)-enantiomer of diphenyl-1-(4-methylbenzothiazole-2-amino)-1-(thiophene-2-yl)-methylphosphonate (**Q-*R***) outperformed its (*S*)-enantiomer (**Q-*S***), demonstrating superior curative, protective, and inactivation activities. Advanced methods, including fluorescence spectroscopy (FS), ITC, MST, MD simulations, and site-directed mutagenesis of the CP protein, identified Arg90 as a critical amino acid residue for the binding of these compounds to TMV CP (Figure 6c). This interaction may alter the configuration of CP, thereby disturbing the assembly of TMV [91].

(c) Marine biological sources

**Figure 7:**
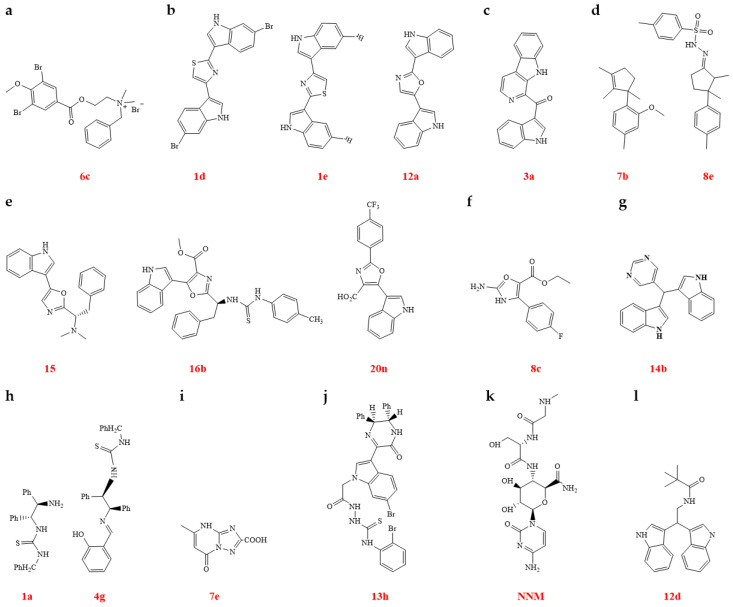
Structures of some derivatives of lead compounds derived from marine natural products or microbial sources with anti-TMV activity that disrupt the assembly of the TMV CP. (**a**) Pulmonarin derivative **6c**, (**b**) nortopsentin analogues **1d**, **1e**, and **12a**, (**c**) a pityriacitrin alkaloid analogue **3a**, (**d**) sesquiterpene derivatives **7d** and **8e**, (**e**) almazoles C, D derivatives **15**, **16b**, and **20n**, (**f**) a polycarpine analogue **8c**, (**g**) a bis (indole) alkaloid barakacin derivative **14b**, (**h**) chiral diamine compounds containing the core structures of harmine and tetrahydroharmine **1a** and **4g**, (**i**) essramycin derivatives **1a**, **4g**, and **7e**, (**j**) a hamacanthin derivative **13h**, (**k**) ningnanmycin, and (**l**) a streptindole derivative **12d**.

Marine natural products from marine animals, marine plants, and marine microorganisms are proving to be crucial in the discovery of novel biocidal compounds. Pulmonarins were isolated from the sub-Arctic ascidian *Synoicum pulmonaria*, and their alkaloid analogues were synthesized and assessed for efficacy against TMV. The majority of these compounds showed anti-TMV effects superior to that of ribavirin. Among them, compounds **6a**, **6c**, and **6n** also showed greater inactivation of TMV in vivo when compared with NNM (Figure 7a). Compound **6c** was shown to hinder virus assembly by binding to TMV CP. MD simulations supported the finding that this compound engaged in hydrogen bonding with the CP [92]. Nortopsentin analogues were synthesized and evaluated for their ability to combat TMV. Compounds **1d**, **1e**, and **12a** demonstrated antiviral activity superior to ribavirin (Figure 7b). Mechanistic studies revealed that compound **1e** induced CP disk aggregation, which facilitated virus clustering. This aggregation may disrupt viral movement within plants and negatively impact the infection process (Table 3) [12]. Pityriacitrin was first isolated from the marine bacterium *Paracoccus* sp. Its alkaloid analogues were then synthesized and tested for efficacy against TMV. Compounds **3a**, **3e**, **8f**, **8g**, and **9g** exhibited strong antiviral effects in vivo, surpassing those of ribavirin. Compound **3a** was found to have a high affinity for binding to TMV CP, thereby disrupting the virus assembly process (Figure 7c, Table 3) [93]. A range of marine sesquiterpene derivatives were synthesized and assessed for their effectiveness against TMV. Compounds **7b** and **8e** exhibited significantly higher antiviral activities in vivo, surpassing that of ribavirin. Specifically, compound **8e** was found to hinder the formation of the 20S protein disk by targeting TMV CP, thereby preventing the assembly of TMV particles (Figure 7d, Table 3) [94]. This study offers a fresh perspective on the development of new antiviral drugs using marine sesquiterpenes. Almazoles C and D, both marine natural products, were chosen as the lead compounds for the synthesis of almazole derivatives, which were then tested for their effectiveness against TMV. The results showed that both almazole C and D, as well as their derivatives, demonstrated significant anti-TMV activity. Almazole alkaloids are a group of oxazole alkaloids isolated from red algae. Almazole D (**7**) and almazole C (**15**) demonstrated anti-TMV activity comparable to that of ribavirin. Several derivative compounds showed even greater anti-TMV activity in vivo than ribavirin. Mechanistic studies revealed that certain compounds could disrupt TMV assembly by inducing polymerization of the 20S CP disk. MD results confirmed that specific candidate compounds could bind to amino acid residues on the TMV CP through hydrogen bonds, leading to alterations in CP structure or function (Figure 7e) [95].

A range of new polycarpine analogues was created and tested for their effectiveness against TMV. In vivo studies showed that compounds **4**, **6f**, and **8c** had significant antiviral effects, surpassing those of NNM (Figure 7f). These findings suggest that compound **8c** may prevent the formation of 20S CP disks by binding to TMV CP [96]. New bis (indole) alkaloids were discovered in marine organisms and microorganisms, and these compounds exhibited a wide array of biological effects, such as antiviral, antitumor, antibacterial, and anti-inflammatory properties [97]. Derivatives of the bis (indole) alkaloid barakacin were synthesized, and their effectiveness against TMV was assessed. Compound **14b** showed significant antiviral activity in vivo, surpassing that of ribavirin (Figure 7g). A study of its mechanism of action revealed that compound **14b** can disrupt virus assembly by affecting the formation of the 20S CP [98]. A group of chiral diamine compounds was synthesized, containing the central structures of harmine and tetrahydroharmine. Their effectiveness against TMV was tested, and it was found that compounds **1a** and **4g** showed stronger antiviral effects in vivo than NNM. Analysis using TEM and MD suggested that these candidate compounds could hinder virus assembly by binding to the TMV CP, thus disrupting the assembly of CP with RNA of TMV (Figure 7h). The authors of this article suggested that extracting key core structures from natural products and making derivative modifications could be a promising approach for developing highly effective antiviral agents for crop use (Table 3) [99]. Essramycin, a triazolopyrimidine natural product with antibacterial activity, was discovered in the culture broth of *Streptomyces* sp. from marine sources [100,101,102]. Spiramycin derivatives were developed, synthesized, and evaluated for their antiviral activity. Most of these compounds showed stronger antiviral effects than ribavirin and NNM. In vivo studies indicated that compounds **7e** and **8f** exhibited higher antiviral efficacy than NNM. Notably, compound **7e** was found to induce aggregation of 20S proteasome subunits, which disrupted virus assembly (Figure 7i, Table 3) [103]. Derivatives based on the bis (indole) alkaloid 6″-debromohamacanthin A were designed, synthesized, and assessed for their antiviral activity. Most of these compounds exhibited stronger antiviral effects than ribavirin. In vivo studies have shown that compounds **1a**, **13e**, **13f**, **13g**, and **13h** had antiviral activities comparable with or greater than NNM. Thermodynamic analysis, MD, and FT techniques revealed that candidate compound **13h** could bind to TMV CP, disrupting the assembly of TMV CP and RNA (Figure 7j, Table 3) [104]. The authors proposed that hamacanthin alkaloids could serve as lead compounds for antiviral agents and are worth developing for innovation in the field of crop protection products.

**Table 3:** Summary of chemical synthetic anti-TMV agents whose lead molecules are derived from marine biological sources.

Antiviral Agents	Mechanisms	Efficacy ^a, b^	References
Nortopsentin derivative **1d**	Induces CP disks aggregation, therefore interfering with viral movement	64.0%	[12]
Pityriacitrin alkaloids derivative **3a**	Binds CP, therefore disrupting the viral assembly	64.0%	[93]
Marine sesquiterpene derivative **7b**	Prevents the formation of 20S protein discs, therefore inhibiting viral particle assembly	53.0%	[94]
Chiral diamine compound **1a**	Binds CP, therefore interfering with viral assembly	73.0%	[99]
Essramycin derivative **7e**	Induces aggregation of the 20S proteasome subunit, therefore disrupting the viral assembly	65.0%	[103]
6″-Debromohamacanthin A derivative **13h**	Interferes the assembly of CP and RNA of virus	59.0%	[104]

^a^ If the specific concentration is not listed in the ‘Efficacy’ column, the concentration is assumed to be 500 μg/mL. ^b^ If the specific mode of action is not listed in the ‘Efficacy’ column, the mode of action is assumed to be curative activity.

(d) Microbial sources

NNM, derived from the fermentation broth of *Streptomyces noursei* var. *xichangensis*, has been extensively researched as an antiviral agent. It is used in China to manage viral and bacterial diseases in crops, as well as fungal diseases in tea plantations (Figure 7j) [105,106]. NNM has the ability to bind to multiple sites on the CP of TMV, interfering with CP assembly. This action can cause a structural change in the CP from disk-like forms to individual units. As a result, when combined with RNA, TMV CP completely loses its ability to cause disease [107]. NNM has undergone extensive development over the years, yet its active components still hold potential for further exploration. Some experts suggest that NNM’s antiviral effects stem from its homologous and multi-component synergistic actions, a viewpoint with which we concur. Due to NNM’s intricate structure, limited research has been conducted on modifying its derivatives. Streptindole, a genotoxic metabolite, was identified and derived from the human intestinal bacterium *Streptococcus faecium* IB 37 [108]. The development of streptindole as an antiviral agent has led to the synthesis and evaluation of several derivatives selected for their antiviral properties. Some compounds, including **4**, **5**, **11**, **12c**, **12d**, **13d**, and **13i**–**13l**, demonstrated higher in vivo anti-TMV activity than ribavirin. Compound **12d** showed particularly promising antiviral activity and was further studied for its mode of action (Figure 7k). Investigations into the mechanism of action showed that compound **12d** could interfere with the structure of TMV CP by forming hydrogen bonds, inhibiting virus particle assembly [11]. This study provides valuable insights into identifying natural product-derived lead compounds with inhibitory activity toward plant viruses.

(e) Chemical synthesis

**Figure 8:**
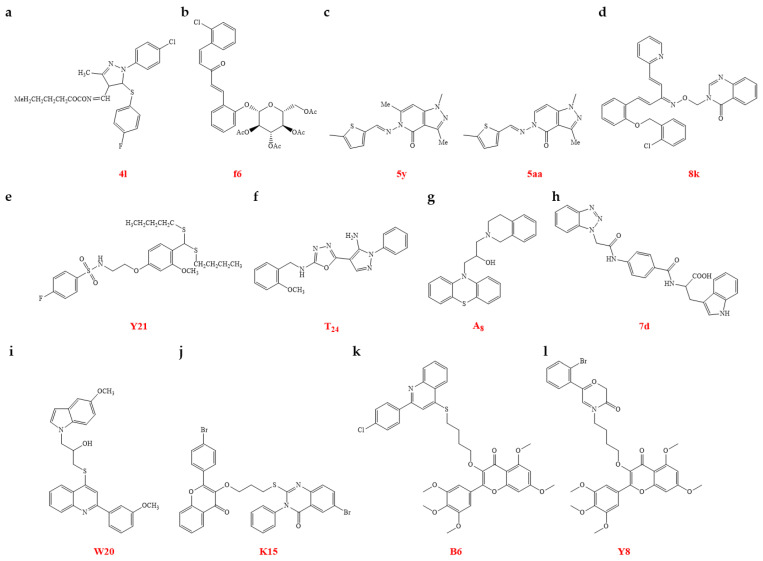
Structures of some compounds from chemical synthesis with anti-TMV activity that disrupt the assembly of the TMV CP. (**a**) A pyrazole derivative containing oxime ester group (**4l**), (**b**) a glucopyranoside derivative with a 1,4-pentadien-3-one functional group (**f6**), (**c**) pyrazolo[3,4-d]pyrimidine compounds featuring a Schiff base (**5y** and **5aa**), (**d**) a penta-1,4-diene-3-one oxime ether derivative linked to a quinazolin-4(3H)-one framework (**8k**), (**e**) a dithioacetal derivative containing a sulfonamide moiety (**Y21**), (**f**) a 1-phenyl-5-amine-4-pyrazole thioether derivative with a 1,3,4-oxadiazole moiety (**T_24_**), (**g**) a phenothiazine derivative (**A_8_**), (**h**) a bisamide-decorated benzotriazole derivative (**7d**), (**i**) an indole derivative containing a quinoline moiety (**W20**), (**j**) a flavonol derivative containing a quinazolinone moiety (**K15**), (**k**) a myricetin derivative containing a thioether quinoline moiety (**B6**), and (**l**) a myricetin derivative containing a benzoxazinone moiety (**Y8**).

The chemical synthesis of pyrazole derivatives containing oxime ester groups were conducted, and evaluated for their anti-TMV activity. Compounds **4l** and **4m** showed higher antiviral activities in vivo than NNM at a concentration of 500 µg/mL, with antiviral effects of 62.0% ± 8.0 and 60.0% ± 8.0, respectively, compared with NNM, with a corresponding antiviral activity of 56.0% ± 4.0. The pyrazole derivative **4l** was found to specifically bind to CP, as observed through FS, thereby inhibiting the proliferation and spread of the virus (Figure 8a, Table 4) [109]. Glucopyranoside derivatives were synthesized and tested for their anti-TMV activity. The results of the bioassays showed that certain compounds demonstrated strong antiviral effects in vivo. Subsequent evaluation of the compounds’ activities against TMV in vivo revealed that compounds **f5**, **f6**, **f8**, **f10**, **f13**, **f14**, **f20**, **f24**, **f26**, and **f28** effectively deactivated TMV, with EC_50_ values ranging from 52.9 to 67.4 μg/mL, compared with ribavirin (145.1 µg/mL). Further investigation into the mode of action indicated that compound **f6** specifically binds to TMV CP, as confirmed by FS, ITC, and MST assays. This suggests that the synthesized glucopyranoside derivative, which contains a 1,4-pentadien-3-one moiety, disrupts the virus assembly by binding to the CP (Figure 8b, Table 4) [110].

A group of pyrazolo[3,4-d]pyrimidine derivatives containing a Schiff base component was synthesized and tested for effectiveness against TMV. Specifically, compounds **5y** and **5aa** demonstrated a strong ability to deactivate TMV, with EC_50_ values of 70.3 and 53.7 µg/mL, respectively; these compounds were significantly better than ribavirin (150.5 µg/mL), with compound **5aa** also outperforming NNM (EC_50_ = 55.4 µg/mL). Both compounds showed potent antiviral activity and exhibited a high affinity for binding to TMV CP in vitro. The findings suggest that pyrazolo[3,4-d]pyrimidine derivatives have potential as anti-TMV agents (Figure 8c, Table 4) [111]. Penta-1,4-diene-3-one oxime ether and derivatives of quinazolin-4(3H)-one exhibit a wide range of biological activities of potential in agriculture and have been developed as pesticides. Penta-1,4-diene-3-one oxime ether derivatives containing a quinazolin-4(3H)-one scaffold were synthesized and tested for their anti-TMV activities. Several compounds (**8c**, **8j**, and **8k**) showed strong binding to TMV CP through the MST assay and MD analysis. Compounds **8c**, **8j**, and **8k** also demonstrated significant curative activities against TMV in vivo with EC_50_ values of 138.5, 132.9, and 125.6 µg/mL, respectively, superior to that of NNM (EC_50_ value of 207.3 µg/mL). Arg90 is an active residue known to be targeted by candidate agent **8k** for TMV CP (Figure 8d, Table 4) [112]. Dithioacetal derivatives with a sulfonamide component were synthesized and tested for their effectiveness against TMV. **Y14**, **Y18**, and **Y21** exhibited strong antiviral effects on TMV, with EC_50_ values for curative, protective, and inactivating activities of 183.0 ± 3.2, 252.3 ± 2.6, and 63.8 ± 1.2 µg/mL for **Y14**, 270.6 ± 3.7, 249.7 ± 3.5, and 57.7 ± 1.4 µg/mL for **Y18**, and 329.5 ± 1.5, 269.2 ± 3.7, and 48.1 ± 2.0 µg/mL for **Y21**, respectively; these values were all superior to those of NNM, which had corresponding EC_50_ values of 331.0 ± 2.8, 271.0 ± 2.8, and 77.4 ± 1.3 µg/mL, respectively. Observations from the TEM assay led the authors to speculate that the dithioacetal derivative **Y21** bound strongly to TMV CP, thereby disrupting the assembly of the virus (Figure 8e, Table 4). Meanwhile, the MST analysis combined with CP mutants indicated that some compounds could strongly bind with the CP, thereby interfering with its function or structure [113].

**Table 4:** The summarizing of chemical synthesis antiviral agents with anti-TMV activity that disrupt the assembly of the TMV CP.

Antiviral Agents	Mechanisms	Efficacy ^a, b^	References
Pyrazole derivative **4l**	Binds to CP, inhibiting viral proliferation and spread	62.0%	[109]
Glucopyranoside derivative **f20**	Binds to TMV-CP, disrupting viral assembly	EC_50_ values: 67.4 µg/mL	[110]
Pyrazolo[3,4-d]pyrimidine derivative **5y**	Binds to TMV-CP	EC_50_: 70.3 µg/mL	[111]
Penta-1,4-diene-3-one oxime ether derivative **8k**	Binds to TMV-CP	EC_50_: 125.6 µg/mL	[112]
Dithioacetal derivative **Y14**	Binds to TMV CP	EC_50:_ 183.0 µg/mL	[113]

^a^ If the specific concentration is not listed in the ‘Efficacy’ column, the concentration is assumed to be 500 μg/mL. ^b^ If the specific mode of action is not listed in the ‘Efficacy’ column, the mode of action is assumed to be curative activity.

New thioether derivatives with a 1,3,4-oxadiazole group were created and tested for their effectiveness against TMV. **T_2_**, **T_7_**, **T_9_**, **T_24_**, **T_25_**, and **T_27_** showed a strong ability to deactivate TMV (Figure 8f). New thioether derivatives with a 1,3,4-oxadiazole moiety were synthesized and tested for their ability to combat TMV. Compounds **T_2_**, **T_7_**, **T_9_**, **T_24_**, **T_25_**, and **T_27_** exhibited strong inactivation activity against TMV, with EC_50_ values of 15.7, 15.7, 15.5, 11.9, 12.5, and 16.5 µg/mL, respectively, outperforming NNM (40.3 µg/mL). Atomic force microscopy and TEM studies revealed that **T_24_** could shorten the length of TMV particles and disrupt their rod-shaped structure. MST and MD analyses indicated strong binding between the compound and TMV CP, with a potential action site identified at Glu50 in the TMV CP. The authors suggested that Glu50 may serve as a novel binding site for antiviral agents targeting TMV CP (Table 5) [114]. Novel derivatives of phenothiazine were synthesized, and their effects on TMV were evaluated. Compound **A_8_** showed strong antiviral activity against TMV, with an EC_50_ of 115.7 µg/mL, which was significantly better than the activities of NNM (271.3 µg/mL) and ribavirin (557.5 µg/mL). The study on the mechanism of action suggested that the compound disrupted the self-assembly of TMV by binding to the TMV CP (Figure 8g, Table 5) [115]. New benzotriazole derivatives with novel bisamide groups were synthesized and tested for their effectiveness against TMV. Compound **7d** demonstrated significant antiviral activity (EC_50_ = 157.6 µg/mL), outperforming ribavirin (EC_50_ = 442.1 µg/mL) (Figure 8h). The combination of TEM and MST analysis revealed that compound **7d** had the ability to disrupt the assembly of TMV by interacting with the TMV CP (Table 5) [116]. The authors believed in the potential of bisamide-decorated benzotriazole derivatives for the development of antiviral agents. They synthesized and evaluated four series of compounds, namely indole derivatives containing quinoline structures, flavonol derivatives containing quinazolinone, and myricetin derivatives containing either thioether quinoline or benzoxazinone moieties, for their anti-TMV activity. Some of the target compounds showed high antiviral activity compared with NNM. Specifically, of the indole derivatives containing quinoline structures, **W7**, **W18**, **W20**, and **W21** exhibited strong antiviral activity with EC_50_ values of 86.6, 137.3, 84.4, and 168.4 µg/mL, respectively, in terms of curative activity, which were superior to NNM (205.1 µg/mL). MST analysis revealed that **W20** had a strong binding affinity for the TMV CP, with a K_d_ of 0.00519 µmol/L, which was superior to that of NNM (1.65 µmol/L) (Figure 8i, Table 5) [117]. The flavonol derivatives containing quinazolinone, particularly **K5** and **K17**, exhibited significant antiviral activity, with EC_50_ values of 120.6 and 177.8 µg/mL, respectively, were more potent than NNM, which had an EC_50_ value of 207.0 µg/mL (Figure 8j) [118]. Compound **B6**, a myricetin derivative featuring a thioether quinoline group, exhibited significant antiviral properties with an EC_50_ of 169.0 µg/mL for curative activity, surpassing the control NNM with an EC_50_ of 227.2 µg/mL. MST analysis revealed that **B6** bound tightly to the TMV CP with a K_d_ of 0.0130 µmol/L, which is much lower than the K_d_ values for myricitrin and NNM (Figure 8k, Table 5) [119]. The myricetin derivative **Y8**, which incorporates a benzoxazinone group, had greater therapeutic and preventative properties against TMV, with EC_50_ values of 236.8 µg/mL and 206.0 µg/mL, respectively, outperforming NNM (372.4 µg/mL and 360.6 µg/mL, respectively). MST data showed that **Y8** had a stronger binding affinity for TMV CP, with a K_d_ of 0.045 µM, compared with NNM (0.70 µM). MD simulations suggested that **Y8** engaged with various amino acids in the TMV CP via non-covalent interactions, affecting the assembly of TMV particles (Figure 8l) [120].

**Figure 9:**
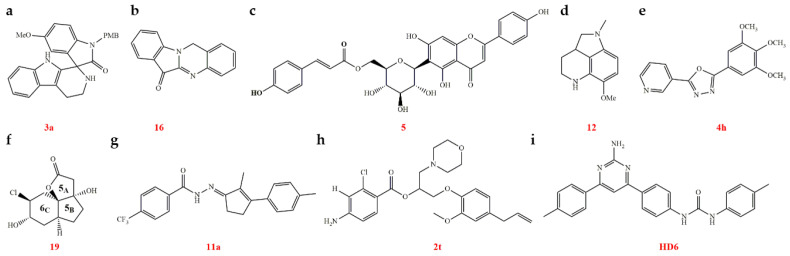
Structures of some chemically synthesized compounds with anti-TMV activity that disrupt the depolymerization of TMV CP, preventing the uncoating process of the virus, binding TMV CP, TMV helicase, or TMV HC-Pro. (**a**) An iheyamine A derivative **3a**, (**b**) a tryptanthrin derivative **16**, (**c**) a flavonoid type candidate compound **5**, (**d**) a dehydrobufotenine derivative **12**, (**e**) an arecoline derivative **4h**, (**f**) a tricyclic spirolactone derivative **19**, (**g**) a laurene derivative containing thiazole, hydrazone, and amide groups **11a**, (**h**) a eugenol derivative candidate compound **2t**, and (**i**) a urea derivative **HD6**.

Research is also being conducted on natural compounds as potential treatments for plant viral infections. Iheyamine alkaloids have been extracted from a marine tunicate found off the coast of Okinawa Island [121]. Iheyamine A and its analogues were synthesized and assessed for their activity against TMV. Among them, compounds **3a**, **3d**, **6p**, and **7a** demonstrated superior in vivo anti-TMV effects, outperforming NNM (Figure 9a). Through a detailed structure–activity analysis, it was found that compound **3a** could bind to the CP, as indicated by MD analysis. These findings suggest that iheyamine A and its derivatives may disrupt TMV CP assembly by binding to it (Table 5) [122].

##### Disrupting the Depolymerization of TMV CP

Tryptanthrin and its analogues were created and tested for their ability to combat viruses. Among them, compounds **3n**, **14**, and **16** showed strong antiviral effects against TMV on *Nicotiana tabacum* var. Xanthi nc, matching or exceeding the performance of ribavirin. Compound **16** was found to inhibit virus assembly by degrading the 20S CP disk into its monomeric state (Figure 9b). The MD analysis indicated that these compounds exhibited stronger interactions with TMV CP (Table 5) [123].

*Clematis lasiandra* is an important medicinal plant. A novel compound, kaempferol 3-*O*-(2″-benzoyl)-β-D-glucopyranosyl-7-*O*-α-L-rhamnopyranoside, was identified in *C. lasiandra*, accompanied by nine previously identified flavonoids. Compounds **2**, **5**, and **6** exhibited considerable antiviral effects against TMV in vivo at a concentration of 500 µg/mL, with inhibition rates of 64.6% ± 2.9, 74.7% ± 1.9, and 82.5% ± 1.9, respectively. Their efficacy was superior to that of NNM, which showed an antiviral effect of 63.8% ± 1.7 (Figure 9c). Candidate compound **5** was found to directly disrupt TMV particles into small fragments, representing a fission phenomenon [124].

Dehydrobufotenine analogues were synthesized and evaluated for their effectiveness against TMV. Compounds **12** and **17** demonstrated strong in vivo antiviral activity, surpassing that of NNM (Figure 9d). Investigation into their mechanism of action suggests that compound **12** may induce fusion and clustering of 20S CP particles. MD simulations revealed hydrogen bonding interactions between the two compounds and the TMV CP (Table 5) [125].

Arecoline was isolated from the betel nut palm *Areca catechu* and is known to be the main bioactive compound [126]. Arecoline possesses diverse biological effects in the medical field, yet studies on its application as an agricultural crop protection compound are limited. Arecoline-based compounds were synthesized and tested for their ability to inhibit TMV. The candidate compounds **4a**, **4h**, **4l**, **4p**, **6a**, **6c**, and **6f** showed high antiviral activity, superior to that of NNM (Figure 9e). Among these compounds, candidate compound **4h** induced virus fragmentation by acting on TMV CP (Table 5) [127].

##### Preventing the Uncoating Process of the Viral Particles

Tricyclic spiranoid lactones, key natural polycyclic molecules with **5A**, **5B**, and **6C** ring junctions, display a spectrum of agricultural bioactivities. Synthesized tricyclic spirolactones were assessed for their antiviral potential. Compounds **14**, **16**, **19**, **23**, and **28** showed exceptional antiviral efficacy against TMV, outperforming NNM. These compounds were effective in cross-linking TMV, inhibiting the virus’s uncoating (Figure 9f). They were capable of cross-linking TMV, thus halting the virus’s uncoating process (Table 5) [128].

##### Binding of TMV CP

Sesquiterpenoid hydrocarbons, sourced from the *Laurencia* red seaweeds, are distinguished by their molecular framework, which incorporates 1-, 2-, and 3-mono-substituted cyclopentenes [129]. Derivatives of the laurene sesquiterpenoid, with thiazole, hydrazone, and amide groups, were synthesized and tested for their effectiveness against TMV. Compounds **5a**–**5c**, **5i**, **5k**, **5l**, **11a**, **11j**, and **12c** demonstrated enhanced antiviral properties in vivo, exceeding the performance of NNM. Compound **11a**, in particular, showed remarkable antiviral efficacy and robust interaction with the TMV CP (Figure 9g, Table 5) [130]. The interaction of the compound with TMV CP could modify the protein’s structure and its functional properties.

#### 2.2.4. Helicase of TMV

##### Binding TMV Helicase

Cytosinpeptidemycin, a secondary metabolite from *Streptomyces ahygroscopicus*, was isolated from soil samples in Tianzhu Mountain, Liaoning, China. It has been shown to effectively inhibit TMV and SRBSDV infections both in vivo and in vitro [130,131,132,133]. The TMV superfamily 1 helicase (TMV-Hel) was cloned, expressed, and purified in *E. coli*. Binding assays using MST and ITC revealed a strong interaction between cytosinpeptidemycin and TMV helicase. MD simulations identified His375 on TMV helicase as the site of interaction, highlighting its importance as a binding site for antiviral agents and suggesting it as a potential target for further investigations [134]. Additionally, strong binding between NNM and TMV helicase was observed, suggesting that NNM may exert a multi-target synergistic effect on TMV [135]. Ribavirin also exhibited strong binding with TMV helicase, indicating different targets compared with those viruses that infect humans [136]. Furthermore, the combination pattern of NNM, cytosinpeptidemycin, ribavirin, and TMV helicase is also worthy of further study.

The presence of multiple viral targets in the interaction between ferulic acid derivatives and TMV CP was also investigated. Derivatives incorporating substituted isopropanolamine moieties were synthesized, and their anti-TMV activity was evaluated. Some candidate compounds displayed high antiviral curative activities and bound strongly to TMV helicase. MD analysis revealed that the candidate compound ***R*-A19** bound to TMV helicase in the binding pocket via multiple hydrogen bonds (Table 5) [137]. Pyrimidine moroxydine derivatives, featuring pyrimidine rings and a moroxydine framework, were synthesized and evaluated for their activity against TMV. One compound, **GLY-15**, effectively inhibited TMV, reducing viral load by 78.3% at a dose of 500 µg/mL. MD simulations suggested that **GLY-15** interacted with both TMV helicase and the CP. The researchers hypothesized that **GLY-15** exerted its antiviral effects by targeting these key sites and enhancing host plant resistance (Table 5) [138]. A targeted virtual screening strategy was utilized to create a novel group of eugenol derivatives, with a focus on inhibiting TMV helicase. Compound **2t** stood out for its significant inhibition of the ATPase activity of TMV helicase, registering a half-maximal inhibitory concentration (IC_50_) value of 141.9 µM. MD simulations indicated that compound **2t** maintained stability and engaged in various interactions within the active site of TMV helicase (Figure 9h) [7]. Phosphonate derivatives featuring a 1,2,3-triazole motif were designed and synthesized as potential inhibitors of TMV helicase. One promising compound, **B17**, showed significant curative activity against TMV in vivo (EC_50_ = 271.5 µg/mL). Mechanistic studies revealed that **B17** effectively inhibited TMV helicase (39.2% inhibition at 300 µM) and exhibited strong binding to the helicase, with a binding affinity of 12.7 µM. MD simulations indicated that **B17** formed stable hydrogen bonds with key amino acid residues (Ala33, Gly10, Gly8, and Glu217) in the active site of TMV helicase [139].

**Table 5:** A summary of chemical synthesis antiviral agents with anti-TMV activity that possess different antiviral mechanisms.

Antiviral Agents	Sources	Mechanisms	Efficacy ^a, b^	References
Thioether derivative **T_24_**	Synthesis	Binds CP on Glu50, therefore causing disassembly of virion	EC_50_: 11.9 μg/mL	[114]
Phenothiazine derivative **A8**	Synthesis	Binds CP, therefore disturbing the formation of CP disks	EC_50:_ 115.7 μg/mL	[115]
Benzotriazole derivative **7d**	Synthesis	Binds CP, therefore disturbing the viral assembly	EC_50:_ 157.6 μg/mL	[116]
Indole derivative **W20**	Synthesis	Binds CP	EC_50_: 84.4 μg/mL	[117]
Myricetin derivative **B6**	Synthesis	Binds CP	EC_50_: 169.0 μg/mL	[119]
Iheyamine A derivative **3a**	Synthesis	Binds CP, therefore interfering with the viral assembly	IC_50_: 162.0 µg/mL (Inactivating activity)	[122]
Tryptanthrin derivative **3n**	Synthesis	Degrades 20S CP discs, therefore inhibiting the viral assembly	49.5%	[123]
Dehydrobufotenine derivative **12**	Synthesis	Binds CP, therefore inducing viral aggregation	57.0%	[125]
Arecoline derivative **4h**	Synthesis	Binds CP, therefore promoting viral fragmentation	77.0%	[127]
Tricyclic spiranoid lactone **19**	Synthesis	Crosslinks vial particles, therefore inhibiting viral uncoating	98.0% at 100 μg/mL (Inactivating activity)	[128]
Laurene derivatives **11a**	Extraction	Binds CP	75.0%	[130]
Ferulic acid derivative **A19**	Synthesis	Binds CP, as well as helicase	EC_50_: 251.1 μg/mL	[137]
Pyrimidine moroxydine derivative **GLY-15**	Synthesis	Binds CP, as well as helicase	73.0% (Viral content)	[138]

^a^ If the specific concentration is not listed in the ‘Efficacy’ column, the concentration is assumed to be 500 μg/mL. ^b^ If the specific mode of action is not listed in the ‘Efficacy’ column, the mode of action is assumed to be curative activity.

#### 2.2.5. TMV Helper-Component Proteinase (HC-Pro)

Urea derivatives containing glucosamine exhibit antiviral activity because they can form numerous stable hydrogen bonds with the target protein [140,141]. A recombinant protein representing the C-terminally truncated TMV HC-Pro (amino acids 307–465) was produced and purified. The MST assay revealed that compound **HD6** had the highest binding affinity for this protein (Figure 9i). MD simulations indicated that **HD6** interacts with the protein at residues Asp121, Asn48, and Tyr38 via several hydrogen-bonding interactions [142].

#### 2.2.6. CMV CP

##### Binding CMV CP

**Figure 10:**
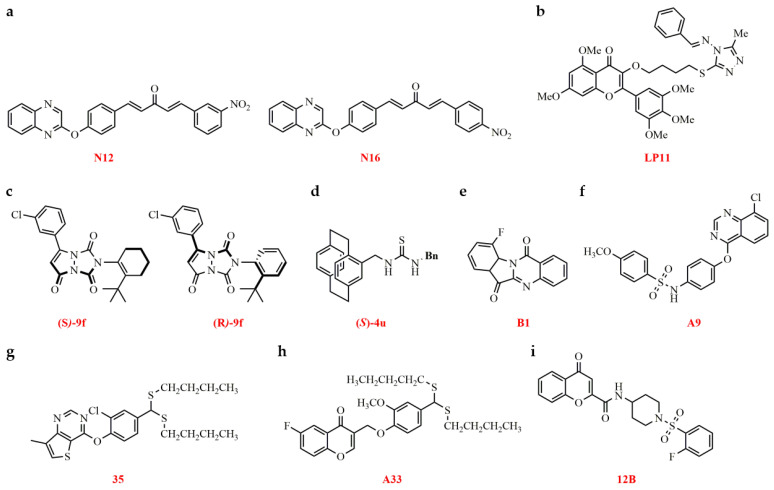
Structures of some compounds with anti-CMV, -PVY, -TeMV, -TSWV, or -SRBSDV activities and their activities. (**a**) Derivatives of 1,4-pentadien-3-one **N12** and **N16**, (**b**) a myricetin derivative **LP11**, (**c**) urazole derivatives (*S*)-**9f** and (*R*)-**9f**, (**d**) a planar chiral thiourea derivative (*S*)-**4u**, (**e**) a candidate compound **B1**, (**f**) a benzenesulfonamide derivative **A9**, (**g**) a thienopyrimidine-containing dithioacetal derivative **35**, (**h**) a chromone derivative containing dithioacetals **A33**, and (**i**) a chromone derivative containing sulfonamide derivative **12B**.

Several 1,4-pentadien-3-one derivatives were synthesized and evaluated for their ability to inhibit CMV. Several of these compounds demonstrated potent antiviral activity. MST experiments revealed that compounds **N12** and **N16** displayed a strong binding affinity for the CMV CP (Figure 10a). MD simulations, supported by site-directed mutagenesis studies, indicated that these compounds interact with key amino acid residues (Ile210, Thr69, and Ser213) on the CMV CP (Table 6). These findings provide valuable insights for the development of novel anti-CMV therapeutics [143].

##### Binding CMV-2b Protein

Myricetin analogues were synthesized and evaluated for their antiviral activity against CMV, with the LP series exhibiting notable potency. Several compounds, including **LP4**, **LP11**, **LP13**, and **LP20**, exhibited strong binding to the 2b protein, as measured by MST and ITC, with K_d_ values of 1.39, 0.880, 1.52, and 1.77 µM, respectively. Among them, **LP11** showed the highest binding affinity for the CMV 2b protein, with a K_d_ of 1.19 µM. Further studies revealed that **LP11** binds to Leu15 and Met18 in the N-terminal region of CMV-2b, as confirmed by mutation experiments (Figure 10b, Table 6). In vitro testing on *Nicotiana benthamiana* plants demonstrated that **LP11** effectively inhibited CMV infection and significantly reduced viral titers [144].

#### 2.2.7. PVY CP Through Binding Between CP and the Antiviral Agent

Research showed that Arg191 (R191) in the CP is a pivotal site for the movement of the PVY viral particle between cells. The compound **-3j** (***S***), synthesized with a high yield of up to 99% and a highly chemo-selective ratio (>99:1 er in most instances) through a carbene-catalyzed [3+4] cycloaddition, exhibited potent anti-PVY properties. It demonstrated curative, protective, and inactivating activities of 61.0% ± 3.8, 63.2% ± 4.0, and 85.4% ± 4.4, respectively, outperforming ribavirin, which had effects of 45.5% ± 2.4, 48.2% ± 1.9, and 63.5% ± 2.1, respectively. Compound **-3j** (***S***) is capable of binding to PVY CP at R191, thereby disrupting viral particle assembly and affecting the spread of infection (Table 6) [145].

Furthermore, research teams in China have been investigating advances in plant antiviral agents targeting PVY CP. For instance, several multifunctional urazole derivatives featuring a stereogenic C-N axis were synthesized, and their efficacy against PVY was assessed. A number of these axially chiral compounds with enriched enantiomers showed potent anti-PVY effects. The compound (***R***)-**9f**, in particular, displayed outstanding curative properties against PVY, with an EC_50_ of 224.9 µg/mL, surpassing the performance of NNM, which had an EC_50_ of 234.0 µg/mL (Figure 10c). Investigations into the mechanism of action revealed that the compounds’ axially chiral structures engaged differently with PVY CP, leading to changes in the protein’s conformation. The (***S***)-**9f** variant displayed a single carbon-hydrogen bond and one π-cation interaction with PVY CP. On the other hand, the (*R*)-enantiomer of **9f** formed three hydrogen bonds, linking the carbonyl groups to Arg157 and Gln158 residues on PVY CP (Table 6). The researchers suggested that axial chirality is a crucial factor in combating plant viruses, potentially facilitating the creation of new, environmentally friendly antiviral agents with enhanced optical purity due to their axial chirality [146]. Planar chiral thiourea compounds were produced via a pivotal *N*-heterocyclic carbene-catalyzed nitrile formation reaction and assessed for their efficacy against PVY. The compounds’ absolute configurations correlated with potent antiviral properties. Notably, the compound (***S***)-**4u** showed exceptional therapeutic potential against PVY, with an EC_50_ value of 349.3 µg/mL (Figure 10d). MD analysis indicated that the planar chiral target compounds could bind to PVY CP, thereby altering the configuration of the CP (Table 6) [147].

#### 2.2.8. PVY VPg Through Binding Between VPg and the Antiviral Agent

Results from MST and ITC experiments revealed that compound **B1** had a robust affinity for PVY VPg, with K_d_ values of 0.690 µmol/L (MST assay) and 4.01 µmol/L (ITC assay), respectively. Additionally, compound **B1** demonstrated significant binding to the VPg proteins of three additional potyviruses. MD simulations suggested that compound **B1** interacts with VPg through a hydrogen bond involving Asn121 (N^121^) (Figure 10e). An assay using the PVY VPg^N121A^ mutant indicated the absence of specific binding between B1 and the mutated protein (Table 6) [148].

#### 2.2.9. TeMV VPg Through Binding Between VPg and the Antiviral Agent

Innovative benzenesulfonamide analogues were created, and their antiviral properties were assessed in vivo and in vitro. The MST test revealed that certain compounds with potent antiviral effects, namely **A4**, **A6**, **A9**, **A16**, and **A17**, exhibited a significant binding preference for TeMV VPg. MD simulations implied that these compounds interacted with TeMV VPg at the Lys121 residue (Figure 10f, Table 6) [149].

#### 2.2.10. N Protein of TSWV Through Binding Between N Protein and the Antiviral Agent

Dithioacetal compound derivatives containing thienopyrimidine were synthesized and evaluated for their antiviral activity against TSWV. Compound **35** demonstrated significant efficacy, with cure, protection, and inactivation rates of 63.0%, 56.6%, and 74.1%, respectively. Its EC_50_ values for protection and inactivation were 252.8 µg/mL and 113.5 µg/mL, respectively, outperforming NNM (284.8 µg/mL and 144.7 µg/mL, respectively) and xiangcaoliusuobingmi, a candidate plant immune activator drug (624.9 µg/mL and 300.0 µg/mL, respectively) (Figure 10g, Table 6) [150]. Innovative chromone-based dithioacetal compounds were created and tested for their ability to combat TSWV. The two compounds, **A32** and **A33,** demonstrated potent in vivo antiviral effects at a 500 µg/mL dose, with efficacy rates of 56.8% ± 4.4 and 55.0% ± 5.0, respectively, outperforming NNM (54.5% ± 4.3) and ribavirin (41.2% ± 5.4). The N protein was cloned, produced in transgenic *E. coli*, and then purified. MST assays showed that compound **A33** showed affinity for the N protein. MD simulations suggested that compound **A33** formed multiple interactions with the N protein (Figure 10h, Table 6) [151]. Several chromone derivatives containing a sulfonamide group were synthesized and tested for their antiviral activity against TSWV. Among them, compound **12B** exhibited significant in vivo antiviral efficacy at a concentration of 500 µg/mL, with an activity rate of 37.2% ± 4.5, outperforming the novel antiviral xiangcaoliusuobingmi (34.0% ± 2.9) (Figure 10i). The MST assay revealed that compound **12B** exhibited a strong binding affinity with the N protein, with a K_d_ of 5.02 µM. MD simulations suggested that compound **12B** formed multiple chemical interactions with the N protein, disrupting its oligomeric structure and impairing its function (Table 6) [152]. Alkaloids derived from seeds of the Mongolian medicinal plant *Thermopsis lanceolata* were extracted, with one compound showing a distinctive dimeric structure typical of quinolizidine alkaloids and exhibiting strong anti-TSWV activity [153]. Furthermore, matrine-type alkaloids from the roots of *Sophora tonkinensis* showed significant anti-TSWV effects by inhibiting the expression of the *N*, *NSs*, and *NSm* genes of TSWV, both in vivo and in vitro [154]. We believe that the inhibition of multiple viral component gene expressions by matrine-type alkaloids is a phenomenon observed after the compounds suppress the virus. The specific antiviral mechanism, however, requires further investigation.

#### 2.2.11. SRBSDV P9-1 Through Binding Between P9-1 and the Antiviral Agent

Sequence analysis of the non-structural P9-1 protein revealed that its C- and N-terminal regions were highly conserved, with a hypervariable segment between positions 131 and 160. Recombinant proteins were produced in *E. coli*, including the wild-type (WT-His-P9-1) and mutant proteins, namely a 23-residue C-terminal deletion (TR-ΔC23-His-P9-1), a 6-residue N-terminal deletion (TR-ΔN6-His-P9-1), and a mutant protein with a serine substitution at position 138 (MU-138-His-P9-1). Fluorescence titration and surface plasmon resonance assays revealed that dufulin binds to WT-His-P9-1 with micromolar affinity, suggesting that the C-terminal 23 amino acids were critical for binding (Table 6) [155].

**Table 6:** The summarizing of chemical synthesis antiviral agents with anti-CMV, -PVY, -TeMV, -TSWV, or -SRBSDV activities and their activities.

Antiviral Agents	Mechanisms	Efficacy ^a, b^	References
1,4-pentadien-3-one derivatives **N12** and **N16**	Binds CMV-CP	Dissociation constants: 0.071 and 0.11 μM	[143]
Myricetin derivative **LP11**	Binds CMV-2b	Kd values: 0.88 μM	[144]
**-3j** (***S***)	Binds PVY-CP	61.0%	[145]
Urazole derivative (***R***)-**9f**	Binds PVY-CP	EC_50_: 224.9 μg/mL	[146]
Thiourea compound ((***S***)-**4u**)	Binds PVY-CP	EC_50_: 349.3 μg/mL	[147]
Tryptanthrin derivative **B1**	Binds PVY VPg	Kd values of 0.690 μmol/L (MST) and 4.01 μmol/L (ITC)	[148]
Benzenesulfonamide derivative **A6**	Binds TeMV-VPg	Kd value: 0.034 μM	[149]
Dithioacetal compound **35**	Binds TSWV-N	EC_50_: 252.8 μg/mL (Protective activity) and 113.5 μg/mL (Inactivating activity)	[150]
Chromone derivative **A32**	Binds TSWV-N	56.8%	[151]
Sulfonamide derivative **12B**	Binds TSWV-N	EC_50_: 80.5 μg/mL	[152]
Dufulin	Binds SRBSDV-P9-1	Kd value: 3.26 μm	[155]

^a^ If the specific concentration is not listed in the ‘Efficacy’ column, the concentration is assumed to be 500 μg/mL. ^b^ If the specific mode of action is not listed in the ‘Efficacy’ column, the mode of action is assumed to be curative activity.

## 3. New Trends of Antiviral Active Substances Targeting Viral Components and Their Mode of Action

### 3.1. Development of Screening Methods for Anti-Plant Virus Agents

The purification of CMV particles involved amine labeling, followed by an assessment in a binding assay to screen for potential antiviral agents [156]. This approach not only improves the speed of drug screening but also reduces associated costs.

Up to now, infectious viral clones, such as TMV, CMV, and PepMoV, which are often tagged for easy observation or for facilitating subsequent experiments, have been developed and used to evaluate the activity and study the mechanisms of plant antiviral agents. The approach involves inserting genes encoding green fluorescent protein (GFP) and other proteins into the coding regions of viral proteins, enabling GFP expression without disrupting the expression or function of the virus’s native proteins [157,158]. The host for the infectious clone has no additional requirements, maintaining consistency with the wild-type virus. Currently, common plant hosts used in experiments include *N. benthamiana*, *Capsicum annuum*, and others. Reverse-transcription quantitative real-time PCR and enzyme-linked immunosorbent assays are effective methods for detecting activity [159]. Infectious clones serve as a key approach for identifying bioactive substances with antiviral properties in living organisms. For instance, trichoderminol, a novel compound extracted from the ethyl acetate fraction of the culture filtrate of the fungus *Trichoderma albolutescens*, and quassinoids from the Chinese medicinal plant *Brucea javanica* were both identified through this screening method [157,158].

### 3.2. A Chemical Biology Study on the Relationship Between Antiviral Agents and Their Targets in Plant Viruses

Studying the life cycle of plant viruses in host plants has provided a foundation for understanding the mechanisms of antiviral agents, particularly against TMV. These mechanisms can be explored through processes like viral uncoating, assembly of the viral CP and the nucleic acid, and viral protein replication. Methods for preparing viral components are well established, including virus collection from host plants, purification, and in vitro expression and purification of viral proteins. Techniques such as FS, circular dichroism (CD), MST, and ITC are also used to analyze the interactions between antiviral agents and their targets [73,109,156]. For instance, FS and CD have been applied to study TMV RNA, while MST and ITC are used for viral CP and HC-PRO. A TEM method was also developed to investigate the virus assembly and its impact on the physicochemical properties of CP.

The aggregation morphology and function of CP, including the state of the four-layer aggregate disk, were studied using a combination of bioinformatic analysis, prokaryotic expression techniques, and chromatographic separation techniques. This work lays the foundation for understanding the interactions between candidate drug molecules and their targets [160,161].

## 4. Conclusions and Future Prospects

**Figure 11:**
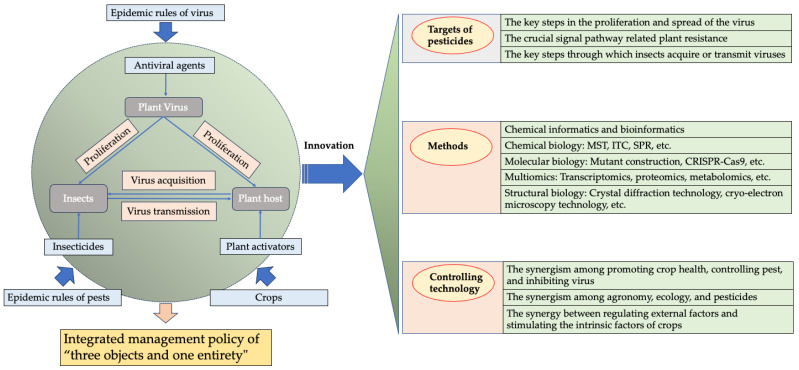
Development of plant virus agents, strategies for controlling plant viral diseases, and future research directions.

In the 25 years from the beginning of this century, China has made significant progress in the development of anti-plant virus agents, including the discovery of lead compounds targeting plant viruses and research into the mechanisms of action of these agents. Simultaneously, Chinese scientists have identified numerous lead compounds with anti-plant viral activity from natural sources and have developed technologies for the modification and synthesis of natural product derivatives [2,3,162]. They have also discovered numerous highly active compounds with anti-plant virus properties and developed crop protection chemicals, such as dufulin and amino oligosaccharins, which offer innovative solutions for controlling viral diseases. These efforts have successfully addressed the issue of viral diseases affecting vegetables, rice, and other crops in China (Figure 11). However, we must also acknowledge the challenges that have arisen in recent years regarding the research and development of antiviral agents. These challenges are as follows:

(1) Limited source, mode of action of antiviral agent: The active substances are derived from a limited range of sources, predominantly plants, marine organisms, and other living organisms. This search for natural compounds has nearly reached its limits, indicating that new approaches are required. Additionally, the research focus has been rather narrow, with most studies centered on TMV, which does not reflect the realities of crop viral disease. Furthermore, the research methods employed are quite similar, primarily relying on the analysis of binding interactions between CPs and candidate drug molecules, along with visualization by electron microscopy.
(2) Limited availability of antiviral agents for farmers: Currently, the limitations of the strategies used mean that there are few anti-plant virus products available, principally dufulin, amino oligosaccharides, and NNM. The main issues are that the efficacy of these antiviral agents in the field control of crop viruses is not particularly impressive, the control costs are high, and their adoption by farmers is challenging. With few products available, it is difficult to meet the demands of agricultural production.
(3) Focus is needed on novel targets, novel methods, and novel controlling technology. Firstly, it is essential to consider the key targets involved in the viral decapsidation, replication, movement, and assembly in the plant hosts and vector insects, as well as the mechanisms of interaction between these viral and host biological macromolecules. Secondly, the research methods and approaches for studying the interaction between targets and active molecules need further expansion. This includes areas such as chemical informatics, bioinformatics, chemical biology, molecular biology, multiomics, and structural biology, which should be actively developed and applied. In addition, there is a need for extensive collection and preparation of transgenic or mutant materials from plants, insects, and other organisms for target research. Thirdly, the approach to the prevention and control of plant viruses should shift from merely inhibiting viral proliferation to regulating it, with a focus on transmission and reducing symptoms. This shift should minimize the incidence of viral diseases, resulting in fewer symptoms and less overall damage to the crop. Finally, it is important to consider the various transmission routes to prevent virus spread by vector insects, examine the effective targeting of these vectors, and investigate agents for preventing insect-mediated transmission. Additionally, coordinating this research avenue with plant activators is crucial to maximizing the effectiveness of treatments. Therefore, we propose the model for plant virus disease control as “three objectives and one overall strategy” (Figure 11).

## Data Availability

The data that support this study are available in the article.
